# Occupational variation in incidence of oropharyngeal cancer in the Nordic countries

**DOI:** 10.1007/s00405-023-08168-6

**Published:** 2023-08-10

**Authors:** Rayan Nikkilä, Antti Mäkitie, Timo Carpén, Johnni Hansen, Sanna Heikkinen, Elsebeth Lynge, Jenny Selander, Ingrid Sivesind Mehlum, Jóhanna Eyrún Torfadottir, Tuula Salo, Eero Pukkala

**Affiliations:** 1 Department of Otorhinolaryngology—Head and Neck Surgery, University of Helsinki, HUS Helsinki University Hospital, P.O. Box 263, 00029 HUS Helsinki, Finland; 2 Finnish Cancer Registry,, Institute for Statistical and Epidemiological Cancer and Research, Helsinki, Finland; 3 Division of Ear, Nose and Throat Diseases, Department of Clinical Sciences, Intervention and Technology, Karolinska Institutet and Karolinska Hospital, Stockholm, Sweden; 4 Research Program in Systems Oncology, Faculty of Medicine, University of Helsinki, Helsinki, Finland; 5 Department of Pathology, University of Helsinki, HUS Helsinki University Hospital, Helsinki, Finland; 6 Danish Cancer Society Research Center, Copenhagen, Denmark; 7 Nykøbing Falster Hospital, University of Copenhagen, Nykøbing Falster, Denmark; 8 Institute of Environmental Medicine, IMM Karolinska Institutet, Stockholm, Sweden; 9 National Institute of Occupational Health (STAMI), Oslo, Norway; 10 Institute of Health and Society, University of Oslo, Oslo, Norway; 11 Icelandic Cancer Registry, Reykjavik, Iceland; 12 Centre of Public Health Sciences, Faculty of Medicine, University of Iceland, Reykjavik, Iceland; 13 Cancer and Translational Medicine Unit, Medical Research Unit, University of Oulu, Oulu University Hospital, Oulu, Finland; 14 Oral and Maxillofacial Diseases, University of Helsinki, and Haartman Institute, Helsinki, Finland; 15 Health Sciences Unit, Faculty of Social Sciences, Tampere University, Tampere, Finland

**Keywords:** Head and neck cancer, Oropharyngeal cancer, Occupational risk, OPC, Oropharynx

## Abstract

**Purpose:**

Evaluate the occupational variation in incidence of oropharyngeal cancer (OPC).

**Methods:**

We calculated standardized incidence ratios (SIRs) of OPC in occupational categories in the Nordic countries relative to the entire national populations. The data covered 6155 OPC cases.

**Results:**

Among men high risk of OPC was observed, among else, in waiters (SIR 6.28, 95% CI 4.68–8.26), beverage workers (SIR 3.00, 95% CI 1.72–4.88), and artistic workers (SIR 2.97, 95% CI 2.31–3.76). Among women high risk of OPC was observed in waiters (SIR 2.02, 95% CI 1.41–2.81) and packers (SIR 1.73, 95% CI 1.07–2.64). The lowest SIRs were observed in female gardeners (SIR 0.27, 95% CI 0.12–0.51) and male farmers (SIR 0.30, 95% CI 0.25–0.35).

**Conclusion:**

The 20-fold variation in incidence of OPC between occupations needs further investigation in studies with detailed information on occupational and non-occupational risk factors.

## Introduction

A dose–response relationship has traditionally been observed between tobacco and alcohol exposure and the appearance of squamous cell carcinoma in the upper aerodigestive tract [1, 2]. Conversely, it has been reported that a fruit and vegetable-rich diet contributes to a lower risk of oral and pharyngeal cancers [3]. Despite a decrease in tobacco consumption, several countries have witnessed an increase in the incidence of oropharyngeal cancer (OPC) over the last decades [4, 5]. Inherent in much of the literature available is a consensus that this increase in OPC has been driven largely by a surge in oral human papilloma virus (HPV) infection, the virus strongly associated with the occurrence of cervical cancer [6]. Globally reported prevalence rates of HPV-driven OPC vary significantly across regions from 0 to 85% [7, 8]. Contraction of HPV occurs primarily via sexual contact, and oral-genital contact can lead to HPV transmission to the oropharyngeal mucosa [9]. Although several strains of HPV exist, the overwhelming majority of HPV-related OPC cases are caused by HPV16 [6].

Several substances, such as formaldehyde, wood and cement dust, coal particles and asbestos, have also been linked to an increased risk of oral and pharyngeal cancers [10–12]. Exposure to high levels of these substances in certain occupational activities may contribute to the development of these cancers in workers. Indeed, studies have reported an association between oral and pharyngeal cancers and numerous occupations, such as construction, painting, and carpentry [11, 12]. However, the contribution of workplace hazards specifically to OPC, the literature available is limited.

We aim to describe the variation in OPC incidence across occupations in the Nordic countries (Denmark, Finland, Iceland, Sweden, and Norway) and discuss the differences observed. To the best of our knowledge no other large-scale study has reported on the incidence of OPC among different occupations. The identification of occupational settings at risk of OPC may contribute to the inception of more targeted occupational health strategies.

## Materials and methods

### Population

The Nordic Occupational Cancer Study (NOCCA) is based on a cohort of 14.9 million people aged 30–64 years and 45 years of cancer incidence data, from 1961 to 2005, linked to occupational categories for all the five Nordic populations of Denmark, Finland, Iceland, Norway, and Sweden [13]. For the NOCCA project, the unique personal identity codes used in all Nordic countries were used to link data from various registries.

### Occupational data

The NOCCA project used occupational data from national censuses and did not conduct its own primary data collection. The original census data retrieval was conducted using several different questionnaires in national languages since the 1960s, which were centrally coded and computerized by the national statistical offices, and partly also by combining data from other databases. Coding of the occupations, which varied among countries, was based on free text information on education, occupation, industry, and name and address of employer at the time of the census. The censuses were carried out in Denmark in 1970, in Finland in 1970, 1980, and 1990, in Iceland in 1981, in Norway in 1960, 1970, and 1980, and in Sweden in 1960, 1970, 1980, and 1990. For each given person, the occupation recorded in the first census the person participated in was recorded. For the NOCCA project, the original occupational codes employed in the censuses were reclassified into 53 occupational categories and one group of economically inactive persons. Information on vital status and emigration was taken from the national population information systems. Person years were calculated from January 1st of the year following the census until date of death, censoring due to immigration, or December 31st of the following years: 2003 in Norway and Denmark, 2004 in Iceland and 2005 in Finland and Sweden (whichever came first).

### Cancer data

The cancer data of this study are collected from the population-based cancer registries in each of the Nordic countries and include all malignancies of the oropharynx, which comprises the base and posterior one-third of the tongue, the tonsils, soft palate, and posterior and lateral pharyngeal walls. A nationwide registration of primary cancer cases was initiated in 1943 in Denmark, in 1953 in Finland and Norway, in 1955 in Iceland, and in 1958 in Sweden. The registries collect data on all new cancer cases notified from hospitals, private clinics, pathology departments and laboratories. All Nordic countries except Sweden also trace missing cases identified via death certificates [14].

### Statistical analyses

Standardized incidence ratios (SIRs) were employed to quantify the risk of OPC across occupational categories relative to the entire national populations. The SIR estimates the risk of developing OPC in a specific occupational category relative to the incidence in the general population. SIR is thus a ratio of observed to expected OPCs. For each country, gender and occupational category, the observed number of cancer cases and person years were stratified into 5-year age categories and 5-year calendar periods. Exact confidence intervals (CI) for SIRs were defined assuming a Poisson distribution of observed number of cases. For data privacy reasons, values are not shown, when less than five cases were reported.

## Results

Among the 14,902,573 study participants 6155 cases of OPC, 4380 in men and 1,775 in women, were diagnosed during the study period between 1961 and 2005 (Table 1). The numbers of observed and expected cases and SIRs for each occupational category are summarized in Table 2.

**Table 1:** Study population and number of oropharyngeal cancer cases stratified by country

Country	Study population (Million)	Follow-up period	Number of oropharyngeal cancers	
			Men	Women
Denmark	2.0	1971–2003	1148	485
Finland	3.4	1971–2005	506	194
Iceland	0.1	1982–2004	10	7
Norway	2.6	1961–2003	760	277
Sweden	6.8	1961–2005	1956	812
Total	14.9		4380	1775

**Table 2:** Observed (Obs) and expected (Exp) numbers of oropharyngeal cancer cases, standardized incidence ratios (SIR), and 95% confidence intervals (CI) among men and women in Denmark, Finland, Iceland, Norway and Sweden during 1961–2005, by occupational category

Occupational category	Men				Women			
	Obs	Exp	SIR	95% CI	Obs	Exp	SIR	95% CI
Technical workers, etc	**269**	**357**	**0.75**	**0.67–0.85**	5	8.8	0.57	0.18–1.32
Laboratory assistants	7	6.1	1.15	0.46–2.37				
Physicians	17	23.2	0.73	0.43–1.17				
Dentists	6	8.8	0.68	0.25–1.48	5	1.8	2.80	0.91–6.52
Nurses					31	32.7	0.95	0.64–1.35
Assistant nurses					46	43.2	1.07	0.78–1.42
"Other health workers"	20	17	1.18	0.72–1.82	**12**	**22.4**	**0.53**	**0.28–0.93**
Teachers	**82**	**135.8**	**0.60**	**0.48–0.75**	48	61.8	0.78	0.57–1.03
Religious workers etc	68	82.9	0.82	0.64–1.04	22	21.4	1.03	0.64–1.56
Artistic workers	**68**	**22.9**	**2.97**	**2.31–3.76**	9	4.2	2.13	0.98–4.05
Journalists	**23**	**11.0**	**2.09**	**1.33–3.14**				
Administrators	227	208.4	1.09	0.96–1.24	15	12.9	1.17	0.65–1.92
Clerical workers	171	155.6	1.10	0.95–1.28	**226**	**178.9**	**1.26**	**1.11–1.44**
Sales agents	**245**	**183.1**	**1.34**	**1.18–1.52**	23	15.6	1.47	0.93–2.21
Shop workers	**170**	**141.1**	**1.20**	**1.04–1.40**	94	98.8	0.95	0.77–1.16
Farmers	**119**	**402.5**	**0.30**	**0.25–0.35**	**26**	**43.8**	**0.59**	**0.39–0.87**
Gardeners	**52**	**103.8**	**0.50**	**0.37–0.66**	**9**	**33.6**	**0.27**	**0.12–0.51**
Fishermen	31	39.4	0.79	0.54–1.12				
Forestry workers	**24**	**68.6**	**0.35**	**0.22–0.52**				
Miners and quarry workers	11	18.5	0.59	0.30–1.06				
Seamen	**112**	**48.7**	**2.30**	**1.91–2.77**				
Transport workers	65	70.9	0.92	0.71–1.17				
Drivers	**263**	**225.2**	**1.17**	**1.03–1.32**				
Postal workers	40	44.8	0.89	0.64–1.22	25	21.4	1.17	0.75–1.72
Textile workers	30	35.4	0.85	0.57–1.21	43	41.6	1.03	0.75–1.39
Shoe and leather workers	12	11.7	1.03	0.53–1.80				
Smelting workers	67	69.6	0.97	0.75–1.23				
Mechanics	327	320.5	1.02	0.92–1.14	13	9.3	1.40	0.75–2.40
Plumbers	29	37.5	0.77	0.52–1.11				
Welders	44	38.6	1.14	0.83–1.53				
Electrical workers	113	119.6	0.95	0.79–1.14	12	7.6	1.58	0.82–2.76
Wood workers	**156**	**214.7**	**0.73**	**0.62–0.85**				
Painters	72	58.3	1.24	0.97–1.56				
“Other construction workers"	143	130.2	1.10	0.93–1.29				
Bricklayers	40	35.6	1.13	0.80–1.53				
Printers	41	39.3	1.04	0.75–1.41	5	4.7	1.06	0.35–2.48
Chemical process workers	44	47.0	0.94	0.68–1.26	5	4.1	1.22	0.40–2.84
Food workers	77	70.3	1.10	0.86–1.37	19	17.9	1.06	0.64–1.66
Beverage workers	**16**	**5.3**	**3.00**	**1.72–4.88**				
Glass makers etc	50	56.4	0.89	0.66–1.17	10	9.0	1.11	0.53–2.04
Packers, loaders, and warehouse workers	**126**	**88.4**	**1.43**	**1.20–1.70**	**21**	**12.2**	**1.73**	**1.07–2.64**
Engine operators	85	92.9	0.91	0.73–1.13				
Public safety workers	**42**	**60.6**	**0.69**	**0.50–0.94**				
Cooks and stewards	**34**	**12.9**	**2.64**	**1.83–3.69**	14	14.5	0.97	0.53–1.62
Domestic assistants					44	54.1	0.81	0.59–1.09
Waiters	**51**	**8.1**	**6.28**	**4.68–8.26**	**35**	**17.3**	**2.02**	**1.41–2.81**
Building caretakers	46	45.8	1.00	0.74–1.34	**113**	**88.4**	**1.28**	**1.06–1.54**
Chimney sweeps	7	2.9	2.38	0.96–4.91				
Hairdressers	16	9.8	1.63	0.93–2.64	15	9	1.66	0.93–2.74
Launderers	7	6.0	1.18	0.47–2.42	6	9.7	0.62	0.23–1.34
Military personnel	37	37.2	0.99	0.70–1.37				
Economically inactive	**376**	**196.3**	**1.92**	**1.73–2.12**	755	809	0.93	0.87–1.00

Values are not shown, if less than five cases were reported. Statistically significant SIRs highlighted in bold

### Men

Among men, the highest SIR of OPC was observed in waiters (SIR 6.28, 95% CI 4.68–8.26), beverage workers (SIR 3.00, 95% CI 1.72–4.88), and artistic workers (SIR 2.97, 95% CI 2.31–3.76). The SIR of OPC in waiters and artistic workers was significantly elevated across all Nordic countries except Iceland, where no cases of OPC were reported for waiters and only one was observed among artistic workers. The SIR for waiters was 7.03 (95% CI 4.16–11.10) in Denmark, 4.97 (95% CI 1.35–12.73) in Finland, 6.64 (95% CI 3.43–11.61) in Norway, and 5.79 (95% CI 3.37–9.27) in Sweden. The SIR for artistic workers was 2.34 (95% CI 1.12–4.30) in Denmark, 2.81 (95% CI 1.21–5.53) in Finland, 2.61 (95% CI 1.19–4.95) in Norway, and 3.26 (95% CI 2.33–4.44) in Sweden.

The incidence of OPC was also significantly elevated in cooks and stewards (SIR 2.64, 95% CI 1.83–3.69), seamen (SIR 2.30, 95% CI 1.91–2.77), journalists (SIR 2.09, 95% CI 1.33–3.14), economically inactive men (SIR 1.92, 95% CI 1.73–2.12), packers, loaders and warehouse workers (SIR 1.43, 95% CI 1.20–1.70), sales agents (SIR 1.34, 95% CI 1.18–1.52), shop workers (SIR 1.20, 95% CI 1.04–1.40), and drivers (SIR 1.17, 95% CI 1.03–1.32).

A reduced incidence of OPC was observed in farmers (SIR 0.30, 95% 0.25–0.35), forestry workers (SIR 0.35, 95% CI 0.22–0.52), gardeners (SIR 0.50, 95% CI 0.37–0.66), teachers (SIR 0.60, 95% CI 0.48–0.75), public safety workers (SIR 0.69, 95% CI 0.50–0.94), wood workers (SIR 0.73, 95% CI 0.62–0.85), and technical workers (SIR 0.75, 95% CI 0.67–0.85).

### Women

Among women, a statistically significant excess incidence of OPC was observed in waiters (SIR 2.02, 95% CI 1.41–2.81), packers, loaders and warehouse workers (SIR 1.73, 95% CI 1.07–2.64), building caretakers (SIR 1.28, 95% CI 1.06–1.54), and clerical workers (SIR 1.26, 95% CI 1.11–1.44). Low SIRs were observed in female gardeners (SIR 0.27, 95% CI 0.12–0.51) and farmers (SIR 0.59, 95% CI 0.39–0.77).

## Discussion

The incidence of OPC has been significantly rising worldwide conditioned by a surge in oral human papilloma virus (HPV) infection [4–6]. Though recently implemented prophylactic HPV vaccines for early adolescent boys and girls are expected to progressively decrease the incidence of HPV-driven OPC, and eventually even eliminate it in the Nordic countries, it is still essential to survey other potential etiological factors [15]. Even though much of the data of this study were collected several decades ago and the exact relevance to contemporary levels of occupational risk factors remains unclear until updated data are available, it provides the opportunity to compare the incidence of OPC across occupational categories. Among men a high excess incidence (SIR > 1.5) was observed in waiters, artistic workers, beverage workers, cooks and stewards, seamen, journalists, and economically inactive individuals. Among women a high excess incidence was discerned in waitresses and packers, loaders and warehouse workers.

The main strength of this study is the extensive population‐based cancer-incidence database and long follow-up. Moreover, quality assessment studies have shown high coverage and accuracy of cancer diagnosis [14]. Regarding occupational classification, validity studies indicate a reasonable accuracy in the Nordic censuses [13]. Our study aimed to unveil occupational categories displaying excess incidence of OPC. The unique personal identity codes used in all Nordic countries guarantee precise linkage between the cancer registries and the census, mortality, and emigration data [16].

While this study delivers interpretable results for further research and public health surveillance, it is essential to draw § This lack of information hampers inferences about associations between exposure to occupational hazards and OPC. Secondly, one could argue that occupational mobility may distort the incidence estimates, as no data on job tenure were included. Fortunately, occupational stability is deemed high in the early decades of follow-up. It was higher among men than women and highest in occupational categories involving long education, such as for physicians or nurses [17]. An additional concern is the insufficient statistical power due to limited number of OPC for some occupational categories, which could undermine the ability to uncover potential meaningful associations.

Our results show an excess incidence of OPC in waiters and waitresses, which corroborates the previous literature which describes waiters being at increased risk of head and neck cancers [18–20]. The increased occurrence of OPC in waiters is most likely due to several types of inhalational workplace hazards. Most importantly, it is well known, that waiters and waitresses can be exposed to environmental tobacco smoking, i.e., passive smoking, which combined with their own alcohol consumption and smoking might explain the six-fold incidence of OPC in this occupational group as compared to average male population [21, 22]. Throughout the Nordic countries and Europe, smoking-free laws in restaurants and bars have been amended since 2000. For instance, since 2000 in Finland, 2004 in Norway, and 2005 in Sweden, restrictions on smoking in restaurants and bars have been implemented. The new laws have led to decreased air nicotine level concentrations [23–27]. Since the current study contains only data up to 2005, potential positive outcomes of these new legislations would not emerge in the results.

Exposure to cooking fumes may also have contributed to the excess incidence of OPC observed, not only in male cooks and stewards, but also in waiters and waitresses. An increased risk of lung cancer [28, 29] has been associated with cooking fumes. Tarvainen et al. previously also described an increased risk of oral and pharyngeal cancers in cooks [11, 30]. In our study, cooks and stewards were coded as one occupational category, which also encompasses flight attendants. However, there were only about 10,000 cabin crew attendants in the Nordic cohort, mainly women, who therefore represent a minority of the almost 80,000 female cooks and stewards in our study. Moreover, previous studies have not uncovered any increased risk of head and neck cancers in cabin crew attendants (increased incidences of skin cancer and female breast cancer have been described) [31–33]. In contrast to men, female cooks and stewards did not show any significant excess in the incidence of OPC in our study (SIR 0.97, 95% CI 0.53–1.62).

Irregularities in lifestyle due to variable working hours and daily habits, such as tobacco and alcohol consumption, may also provide one explanation for the excess incidence of OPC observed not only in the aforementioned occupations, but also for instance in seamen or journalists. HPV infection, a well-known risk factor for OPC, might have also contributed to the increased incidence of OPC among these occupational categories [6]. HPV prevalence in OPC has grown significantly over time worldwide. For instance, in Europe, HPV-positive OPC has increased from 39.7% (95% CI 32.8–47.0) before 2000 to 59.0% (95% CI 30.2–82.7) between 2000 and 2004. [34] Of note, the average latency period from infection to HPV-positive OPC is estimated at around 10 to 30 years, which consequently implies that all HPV-induced OPCs may still not be observable in the subjects of the last census and the SIRs reported for certain occupational categories might have been even higher with longer follow-up [35]. Still, the possibility that increased HPV prevalence could play a role in increasing the incidence of HPV-related OPC in these occupations remains speculative. However, in contrast to seamen, fishermen had a decreased incidence of OPC, indicating that the maritime working environment itself cannot account for the excess incidence. Increased risks of oral and pharyngeal cancers in seafarers, not observable in fishermen, have also been previously described [36].

Artistic workers also showed a high incidence of OPC in our study, comparable to the increased risk of oral cancer previously conveyed (SIR 2.05, 95% CI 1.55–2.66) for this occupational group [30]. Artistic work encompasses different disciplines, such as painting, music, or sculpting, from diverse environments. For instance, painters are typically exposed to numerous chemical compounds. Although they did not show any increased incidence of OPC in our study, it would not be totally unreasonable to think that artistic painters might, to some degree, be exposed to several carcinogens. Concerning male musicians, a significant excess incidence of OPC (SIR 4.36, 95% CI 2.73–6.60) has been previously revealed among this group [37]. Artistic workers are a heterogeneous occupational group, and the excess incidence of OPC observed is unlikely due to a common occupational carcinogenic hazard and most probably mainly due to behavioral risk factors. For instance, a survey study conducted in Finland reported a higher consumption of alcohol among musicians [38]. Likewise, the elevated occurrence of OPC observed in packers, loaders and warehouse workers, particularly among Danish women (SIR 7.17), might ensue from carcinogens contained in fumes arising from operating machines or could simply be due to life habits.

The excess occurrence of OPC observed in Danish male beverage workers is possibly due to the consumption of alcoholic beverages on site. Indeed, our finding is in congruence with the observations of Thygesen et al. [39] who described an elevated risk of oral, digestive, and respiratory tract cancers in Danish brewery workers employed between 1939 and 1963. The authors reported an SIR of 1.65 (95% CI 1.40–1.94) for oral and pharyngeal cancers and hypothesized that the excess incidence was the consequence of high consumption of alcohol. As a matter of fact, until 2001, each Danish brewery worker was allotted six bottles (2.1 L) of beer per day for on-site consumption, which translated into a four-fold consumption of beer, when compared to the average Danish male [39].

Low incidence of OPC was observed in farmers and gardeners. One could speculate that pesticides used in farming could contain carcinogens. Supportive of this hypothesis, a literature review of case–control studies reported a positive association between exposure to pesticides and pharyngeal, laryngeal, and nasal cancers [40]. A significant excess incidence of oral and pharyngeal cancers among farmers has also been described in Italy [41]. The physical activity of farmers and gardeners might perhaps have contributed to the low SIRs in our study. Indeed, evidence, though limited, suggests a link between higher levels of physical activity and lower risk of cancer, including head and neck cancers [42, 43]. The lower-than-average smoking prevalence among Nordic farmers also constitutes a plausible explanation [44, 45]. Though occupational exposure to wood dust has in one study been linked to an increased risk of oropharyngeal cancer [46], wood workers also showed a low incidence of OPC in our study.

Several factors non-related to occupational hazard may affect the incidence rates of cancer, such as differences in the prevalence of etiological factors, diagnostic procedures and cancer reporting measures. As we aimed to describe the occupational variation in incidence of OPC, it was more sensical to compare the observed numbers of OPC in each country with the expected number in the general population of the same country instead of the entire Nordic population. In the Nordic countries, the highest incidence of OPC in the general population is observed in Denmark, likely attributable to the higher Danish rates of smoking and alcohol consumption [47], and the lowest incidence is encountered in Finland. The age-standardized rates (Nordic) for 1960–2005 were 3.0 and 1.1 in Denmark and 1.4 and 0.47 in Finland, for men and women, respectively [48, 49]. The corresponding rates for the whole Nordic region were 2.0 and 0.71 in men and women, respectively. Thus, SIR values reported for Denmark would have been higher and in Finland lower, had the observed numbers of OPC be compared to expected numbers based on the Nordic incidence rates.

## Conclusion

The present study adds to the existing body of literature on occupational cancer and describes an excess occurrence of OPC in certain occupational categories. In particular, waiters carry a high excess incidence of OPC when compared to the general population. A high incidence of OPC was also noted among men in beverage workers, artistic workers, cooks and stewards, seamen, and journalists, and among female packers, loaders and warehouse workers. While the methodology of the study was not designed to estimate dose–response effect of specific occupational exposures, it still highlights the excess incidence of OPC in certain occupational categories. The association between OPC and the specific exposures among at-risk groups need to be further evaluated in studies where detailed occupational exposures and non-occupational risk factors are obtained.
